# Cartilage markers and their association with cartilage loss on magnetic resonance imaging in knee osteoarthritis: the Boston Osteoarthritis Knee Study

**DOI:** 10.1186/ar2314

**Published:** 2007-10-24

**Authors:** David J Hunter, Jiang Li, Michael LaValley, Doug C Bauer, Michael Nevitt, Jeroen DeGroot, Robin Poole, David Eyre, Ali Guermazi, Dan Gale, David T Felson

**Affiliations:** 1 Clinical Epidemiology Research and Training Unit, Boston University School of Medicine, Boston, MA, USA; 2 University of California, San Francisco, San Francisco, CA 94107, USA; 3 Immunological and Infectious Diseases Division, TNO Quality of Life, Leiden, The Netherlands; 4 McGill University, Montreal, QC, Canada; 5 Department of Orthopaedics and Sports Medicine, University of Washington, Seattle, WA 98195, USA; 6 VirtualScopics, Rochester, NY 14625, USA

## Abstract

We used data from a longitudinal observation study to determine whether markers of cartilage turnover could serve as predictors of cartilage loss on magnetic resonance imaging (MRI). We conducted a study of data from the Boston Osteoarthritis of the Knee Study (BOKS), a completed natural history study of knee osteoarthritis (OA). All subjects in the study met American College of Rheumatology criteria for knee OA. Baseline and follow-up knee magnetic resonance images were scored for cartilage loss by means of the WORMS (Whole Organ Magnetic Resonance Imaging Score) semiquantitative grading scheme. Within the BOKS population, 80 subjects who experienced cartilage loss and 80 subjects who did not were selected for the purposes of this nested case control study. We assessed the baseline levels of cartilage degradation and synthesis products by means of assays for type I and II cleavage by collagenases (Col2:3/4C_short _or C1,2C), type II cleavage only with Col2:3/4C_longmono _(C2C), type II synthesis (C-propeptide), the C-telopeptide of type II (Col2CTx), aggrecan 846 epitope, and cartilage oligomeric matrix protein (COMP). We performed a logistic regression to examine the relation of levels of each biomarker to the risk of cartilage loss in any knee. All analyses were adjusted for gender, age, and body mass index (BMI); results stratified by gender gave similar results. One hundred thirty-seven patients with symptomatic knee OA were assessed. At baseline, the mean (standard deviation) age was 67 (9) years and 54% were male. Seventy-six percent of the subjects had radiographic tibiofemoral OA (Kellgren & Lawrence grade of greater than or equal to 2) and the remainder had patellofemoral OA. With the exception of COMP, none of the other biomarkers was a statistically significant predictor of cartilage loss. For a 1-unit increase in COMP, the odds of cartilage loss increased 6.09 times (95% confidence interval [CI] 1.34 to 27.67). After the analysis of COMP was adjusted for age, gender, and BMI, the risk for cartilage loss was 6.35 (95% CI 1.36 to 29.65). Among subjects with symptomatic knee OA, a single measurement of increased COMP predicted subsequent cartilage loss on MRI. The other biochemical markers of cartilage synthesis and degradation do not facilitate prediction of cartilage loss. With the exception of COMP, if changes in cartilage turnover in patients with symptomatic knee OA are associated with cartilage loss, they do not appear to affect systemic biomarker levels.

## Introduction

Osteoarthritis (OA) is characterized by the degeneration of articular cartilage. This results from a direct attack on matrix molecules, resulting in their cleavage, damage to these molecules, and their loss. It is also accompanied by a response of the tissue to this damage which involves enhanced matrix synthesis and turnover. The most direct evidence of pathology is cartilage degradation. A secondary and more indirect indication is cartilage matrix synthesis. The amount of synthesis in relation to degradation may prove of great importance in determining disease progression [1].

The ability to use biochemical markers to predict disease progression and identify patients most likely to progress is a top priority in the future management of OA. Ultimately, it would enable much more rapid assessment of structure-modifying therapies in clinical trials. It may also allow the identification of patients at highest risk of progression, allowing the efficient testing of new treatments. Biochemical markers of OA progression represent a surrogate for structural change which may have advantages over existing methods of measuring structure. Therapeutic development in OA is constrained by the slow progress of structural changes using standard imaging techniques. The development and validation of biochemical markers may accelerate the pace of therapeutic development.

Some recent work on type II collagen has suggested that assays for type II collagen degradation, when used in combination or with markers of collagen synthesis, can distinguish populations with knee OA which exhibit progression of joint damage from non-progressors. The ratio of the type II collagen crosslinking C-telopeptide (CTX-II) to the amino propeptide of type IIA collagen [2] or the ratio of two collagenase-generated cleavage epitopes in the helical region (C1,2C to C2C) [3] each can make this distinction. The results from each of these studies need to be confirmed. But, clearly, these two independent studies point to differences in collagen turnover as being suggestive of disease progression and provide encouragement for future work in this area. Preliminary plain radiographic studies suggest that cartilage oligomeric matrix protein (COMP) may be a useful prognostic marker of disease progression in knee [4-6] and hip [7] OA.

The overarching aim of this investigation was to conduct a study within an existing longitudinal dataset of knee OA with serial knee magnetic resonance imaging (MRI) to evaluate and validate promising biochemical markers, markers that have been reported in either cross-sectional or longitudinal studies to be related to OA or its progression. MRI of the knee has the advantage of covering the whole joint in one examination, meaning that the cartilage defects in the joint can be visualized directly, regardless of their location [8]. Direct visualization of cartilage defects enhances the ability to detect cartilage loss that can be missed using joint space narrowing from plain radiographs [8,9].

More specifically, we assessed the baseline levels of cartilage degradation, synthesis, and turnover products using collagenase-generated C1,2C, and C2C; Col II C-telopeptide (CTX-II assay); C-propeptide of type II collagen; aggrecan 846 epitope; and COMP in a sample of knees with known knee cartilage loss and controls. Our prior hypotheses were that increased levels of cartilage degradation products would be predictive of cartilage loss and that imbalance of cartilage synthesis and degradation would be predictive of cartilage loss.

## Materials and methods

### Study sample

We conducted an analysis of data from the Boston Osteoarthritis of the Knee Study (BOKS), a completed natural history study of knee OA [10]. To be eligible for the study, patients had to have knee pain, aching, or stiffness on most days within the last month and they had to have reported that a physician had told them that they had arthritis in the knee. If they met both of those criteria, they underwent radiography (weight-bearing fluoroscopic posteroanterior, lateral, and skyline views) and if on any of these views they had a definite osteophyte in the symptomatic knee (either tibiofemoral or patellofemoral), they were eligible for the study. In addition, they had to fill out a questionnaire that screened for other forms of arthritis, including rheumatoid arthritis, and information on the use of medications for arthritis was gathered. If a patient screened positive for another form of arthritis or had been receiving medications that were appropriate for rheumatoid arthritis or other forms of arthritis, he or she was excluded. Thus, all subjects in the study had primary clinical knee OA and met American College of Rheumatology criteria for this disorder.

Of 324 subjects who entered the study, 86% completed a full comprehensive follow-up at a later time point (15 and/or 30 months). These comprehensive examinations consisted of an MRI of the more affected knee and a comprehensive set of radiographs, including a semiflexed fluoroscopically positioned posteroanterior radiograph using the method of Chaisson and colleagues [11] and Buckland-Wright [12].

Blood and urine (second morning void) specimens were also obtained at baseline. Specimens were aliquoted and immediately frozen; serum was frozen at -70°C and urine at -20°C. The specimens were stored at a long-term repository (the Biomedical Research Institute, Rockville, MD, USA).

Within the BOKS population, 80 subjects with MRI cartilage loss and 80 subjects without cartilage loss were selected for the purposes of this nested case control study. Cartilage loss was defined as an increase in cartilage score at 30 months from that at baseline. After completion of the assays, 153 participants had data available for all of the biomarker assays. Once the biomarker assay data and MRI data were merged, 137 subjects had complete data (both complete biomarker and longitudinal MRI data) available for analysis. These participants were similar to those from the larger study sample. The institutional review boards of Boston University Medical Center and the Veterans Administration Boston Health Care System approved the baseline and follow-up examinations, and informed consent was obtained from all participants.

### Magnetic resonance imaging

All studies were performed with a Signa 1.5T MRI system (General Electric Comp., Milwaukee, WI, USA) using a phased-array knee coil. A positioning device was used to ensure uniformity of positioning among patients. The imaging protocol included sagittal spin-echo proton density- and T2-weighted images (repetition time [TR] 2,200 milliseconds and time to echo [TE] 20/80 milliseconds) with a slice thickness of 3 mm, a 1-mm interslice gap, 1 excitation, a field of view (FOV) of 11 to 12 cm, and a matrix of 256 × 192 pixels and coronal and axial spin-echo fat-suppressed proton density- and T2-weighted images (TR 2,200 milliseconds and TE 20/80 milliseconds) with a slice thickness of 3 mm, a 1-mm interslice gap, 1 excitation, and the same FOV and matrix.

Cartilage on MRI was scored paired and unblinded to sequence on 14 plates (anterior, central, and posterior femur; anterior, central, and posterior tibia; and medial and lateral patella) using the Whole Organ Magnetic Resonance Imaging Score (WORMS) semiquantitative method [13]. Both cartilage signal and morphology were scored using a 0-to-6 scale: 0 = normal thickness and signal, 1 = normal thickness but increased signal on T2-weighted images, 2 = solitary focal defect of less than 1 cm in greatest width, 3 = areas of partial-thickness defects (less than 75% of the plate) with areas of preserved thickness, 4 = diffuse partial-thickness loss of cartilage (greater than or equal to 75% of the plate), 5 = areas of full-thickness loss (less than 75% of the plate) with areas of partial-thickness loss, and 6 = diffuse full-thickness loss (greater than or equal to 75% of the plate). The intraclass correlation coefficient on agreement for cartilage readings ranged from 0.75 to 0.97.

In WORMS, grade 1 does not represent a morphologic abnormality but rather a change in signal in cartilage of otherwise-normal morphology. Grades 2 and 3 represent similar types of abnormality of the cartilage, focal defects without overall thinning. Therefore, to create a consistent and logical scale for evaluation of cartilage morphologic change, we collapsed the WORMS cartilage score to a 0-to-4 scale in which the original WORMS score of 0 and 1 were collapsed to 0, the original scores of 2 and 3 were collapsed to 1, and the original scores of 4, 5, and 6 were considered 2, 3, and 4, respectively. Cartilage loss was defined as an increase in the score at any subregion compared to baseline in any of the 14 subregions of the knee scored for cartilage in each knee.

We selected subjects who attended the baseline and final visits with an intervisit duration generally more than 30 months. Within the BOKS population, all of the biomarkers mentioned and cartilage loss on serial MRI were available on 137 participants.

### Cartilage biomarkers

The neoepitope resulting from collagenase cleavage of triple-helical type II collagen (Col2:3/4C_long_, also known as C2C) was measured by means of an enzyme-linked immunosorbent assay (ELISA) [14]. It uses a monoclonal antibody that recognizes a sequence near the carboxy terminus of the 3/4 piece.

The C1,2C assay relates to epitopes formed by degradation of type II collagen by collagenase 1, 2, and 3 [15]. Serum concentrations of these degradation products is determined by inhibition ELISA using polyclonal rabbit anti-human antibody.

The C-propeptide of type II collagen (CPII) is cleaved from the procollagen molecule as it forms fibrils. Thus, CPII levels are potentially an index of collagen type II formation and are measured with an ELISA [16].

Aggrecan 846 epitope is present on intact aggrecan molecules (the epitope is associated with chondroitin sulfate chains near the G3 domain) [17]. Aggrecan 846 is measured by ELISA with a mouse monoclonal immunoglobulin M antibody [18].

The C2C, C1,2C, C-propeptide (CPII), and CS 846 commercial assays were obtained from IBEX Technologies Inc. (Montreal, QC, Canada). These have been validated for human studies [19,20].

The intraday (*n *= 20) and interday (*n *= 200) coefficients of variance for each biomarker are, respectively, 10%–17% and 14% for C2C, 5%–14% and 13% for C1,2C, 4%–12% and 12% for CS 846 epitope, and 11%–18% and 16% for CPII. The interassay coefficients of variance for all the assays are in the range of 6.4% to 11.5% [19].

Crosslinked peptides from the C-telopeptide domain of type II collagen (Col2CTx) were quantified by competition ELISA. The assay is based upon a monoclonal antibody, 2B4, which was raised in mice against a conjugated synthetic peptide, EKGPDP [21]. This assay was conducted in the laboratory of author DE. The Col2CTx ratio was Col2CTx/urinary creatinine. The intra-assay and interassay coefficients of variation for the CTx-II/Cr assay were 6% and 13%, respectively [22].

COMP was measured in serum [23] by a solid-phase two-site enzyme immunoassay. It is based on the direct sandwich technique, in which two monoclonal antibodies are directed against separate antigenic determinants on the COMP molecule (intra-assay and interassay variations of less than 5% and a detection limit of less than 0.1 U/L). This is a commercial assay manufactured by AnaMar Medical R&D (Lund, Sweden). With the exception of Col2CTX, all assays were conducted at TNO (Leiden, The Netherlands).

### Statistical analysis

#### Hypothesis 1: increased levels of cartilage degradation products are predictive of cartilage loss

We assessed whether increased levels of each biomarker were predictive of subsequent cartilage loss on knee MRI (ascertained from baseline to visit at 30 months). The six biochemical markers used as predictor variables were Col2:3/4C_long_; C1,2C; Col II C-telopeptide; C-propeptide of type II collagen; aggrecan 846 epitope; and COMP. We used the standardized variable as a predictor to facilitate comparison between the multiple biomarkers.

We firstly assessed the distribution of baseline cartilage scores to ensure that all participants were capable of continued cartilage loss and had not reached a ceiling. We then performed a logistic regression to examine the relation of levels of each logarithmic transformed biomarker to the risk of cartilage loss in any plate. Cartilage loss in a knee was defined as an increase in cartilage score in any of the 14 subregions scored for cartilage in each knee. Considering that the risk profiles of cartilage loss and magnitude of effect of a particular biomarker on cartilage loss may be different between men and women, we first conducted separate analyses for each gender. As the magnitude of effect of biomarkers was similar for men and women, we then performed the analysis adjusting for gender, age, and body mass index (BMI).

#### Hypothesis 2: imbalance of cartilage synthesis and degradation is predictive of cartilage loss

To test this hypothesis, we grouped biomarkers into those that potentially reflect cartilage synthesis (CPII) and those that reflect cartilage degradation (Col2:3/4C_long _[C2C], Col2:3/4C_short _[C1,2C], and Col2CTx). We performed the same analytic approach as above with the predictor variable being a synthesis/degradation marker.

## Results

At baseline, the mean (standard deviation) age was 67 (9) years and 54% were male. The remainder of the demographic characteristics are presented in Table 1. Seventy-six percent of the subjects had radiographic tibiofemoral OA (Kellgren & Lawrence [K&L] grade of greater than or equal to 2) and the remainder had patellofemoral OA. Further descriptive characteristics for the participants are provided according to whether they lost cartilage in any plate during the course of a 30-month follow-up. Compared to those who did lose cartilage at follow-up, there was an over-representation of women and patients with patellofemoral OA (as opposed to TF OA K&L grade of greater than or equal to 2) in participants who did not lose cartilage at follow-up. There was no difference in biomarker levels between the two groups at baseline.

**Table 1:** Baseline characteristics of study population (*n *= 137)

	Whole sample	No cartilage loss in any plate (at follow-up) (*n *= 66)	Cartilage loss in any plate (at follow-up) (*n *= 71)
Age in years, mean ± SD	67 ± 9	66 ± 9	67 ± 10
Males, number (percentage)	74 (54%)	38 (58%)	36 (51%)
Body mass index, mean (SD)	31.43 (5.48)	31.03 (6.08)	31.75 (4.87)
Percentage with K&L grade of ≥ 2	73%	67%	80%
Number of plates with cartilage loss, mean (SD)	0.99 (1.24)	0 (0)	1.92 (1.09)
Mean interval in years between baseline and follow-up scan	2.67	2.68	2.66
Levels of biomarkers, mean (SD)			
COMP	12.5 (2.9)	11.9 (2.9)	13.0 (2.9)
846 epitope	269.3 (154.2)	273.4 (154.6)	265.4 (154.8)
CPII	1,660.9 (598.5)	1,635.9 (623.9)	1,684.1 (577.3)
C1,2C	0.06 (0.03)	0.06 (0.03)	0.06 (0.03)
Col2CTX/Cr ratio	51.77 (63.53)	45.82 (31.81)	57.62 (83.70)
C2C	53.2 (22.9)	57.0 (28.6)	49.7 (15.5)

C2C, collagenase cleavage of triple-helical type II collagen; Col2CTx, crosslinked peptides from the C-telopeptide domain of type II collagen; COMP, cartilage oligomeric matrix protein; CPII, C-propeptide of type II collagen; K&L, Kellgren & Lawrence; SD, standard deviation.

The results of the logistic regression for univariate biomarker predictors with the outcome cartilage loss in any plate are presented in Table 2. With the exception of COMP, none of the other biomarkers was a statistically significant predictor of cartilage loss. For COMP, a 1-standard deviation increase in COMP increased the odds of subsequent cartilage loss 6.09 times (95% confidence interval [CI] 1.34 to 27.67). The C (AUC) statistic for the univariate association was 0.60. After the analysis for COMP was adjusted for age, gender, and BMI, the risk for cartilage loss was 6.35 (95% CI 1.36 to 29.65). The results of the logistic regression for imbalance of biomarker predictors with the outcome cartilage loss in any plate are presented in Table 3. Whereas C2C/CPII approached significance, none of the ratios tested facilitated prediction of cartilage loss.

**Table 2:** Baseline measures of individual standardized cartilage biomarkers and their respective prediction of subsequent cartilage loss on magnetic resonance imaging

	Unadjusted OR (95% CI)	*R*^2^	AUC	*P *value	Adjusted OR^a ^(95% CI)
COMP	6.09 (1.34, 27.67)	0.06	0.60	0.02	6.35 (1.36–29.65)
846 epitope	0.93 (0.51, 1.71)	0.001	0.52	0.82	0.96 (0.52, 1.78)
CPII	1.19 (0.61, 2.32)	0.002	0.53	0.62	1.18 (0.60, 2.32)
C1,2C	0.69 (0.30, 1.59)	0.008	0.55	0.38	0.72 (0.31, 1.68)
Col2CTX/Cr ratio	0.99 (0.65, 1.52)	0.000	0.52	0.97	0.95 (0.61, 1.47)
C2C	0.39 (0.13, 1.13)	0.03	0.58	0.08	0.4 (0.14, 1.17)

^a^Adjusted for age, gender, and body mass index. AUC, area under the curve; C2C, collagenase cleavage of triple-helical type II collagen; CI, confidence interval; Col2CTx, crosslinked peptides from the C-telopeptide domain of type II collagen; COMP, Cartilage Oligomeric Matrix Protein; CPII, C-propeptide of type II collagen; OR, odds ratio.

**Table 3:** Imbalance of baseline measures of standardized cartilage biomarkers and prediction of subsequent cartilage loss on magnetic resonance imaging

	Unadjusted OR (95% CI)	*R*^2^	AUC	*P *value	Adjusted OR^a ^(95% CI)
CTX/CPII	0.72 (0.03, 16.66)	0.001	0.52	0.84	0.53 (0.02, 13.23)
C2C/CPII	<0.001 (<0.001, 1.14)	0.04	0.60	0.05	0.001 (<0.001, 1.46)
C1,2C/CPII	0.24 (0.002, 39.12)	0.003	0.52	0.59	0.31 (0.002, 52.18)
C1,2C/C2C	0.19 (0.02, 2.12)	0.02	0.57	0.18	0.22 (0.02, 2.48)
Col2CTX/Cr ratio/C2C	1.60 (0.31, 8.19)	0.003	0.52	0.57	1.37 (0.26, 7.40)

^a^Adjusted for age, gender, and body mass index. AUC, area under the curve; C2C, collagenase cleavage of triple-helical type II collagen; CI, confidence interval; Col2CTx, Crosslinked peptides from the C-telopeptide domain of type II collagen; CPII, C-propeptide of type II collagen; OR, odds ratio.

## Discussion

Increased COMP levels predict subsequent cartilage loss on MRI, but the association is modest (area under the curve = 0.60). The other biochemical markers of cartilage synthesis, turnover, and degradation do not facilitate prediction of cartilage loss.

Articular cartilage is a multiphasic material with at least two major phases: a fluid phase composed of water and electrolytes and a solid phase composed of chondrocytes together with matrix molecules that include collagen and proteoglycans. The predominant type of collagen is type II, which is found predominantly in cartilage (some also in the vitreous of the eye). It forms the basic fibrillar structure of the extracellular matrix which imparts its tensile strength.

As articular cartilage degenerates in OA, chondrocytes upregulate their biosynthetic activities, including type II collagen, as if to compensate for this damage. Only after secretion, as the molecules reach the extracellular space, are the non-helical domains at the end (the amino-terminal type II and carboxy-terminal type II procollagen propeptides [PIINP and PIICP, respectively]) cleaved from the helical domain.

The C-propeptide content and release from the cartilage are directly correlated with collagen synthesis [24]. In OA, a variant form of type II collagen is produced in which the N-propeptide contains an additional gene product of exon 2 [25]. This is type IIA collagen and it represents a form found in development but apparently not in healthy adult cartilage. The remainder of the collagen is called type IIB and lacks the exon 2 product. By immunoassay, it is possible to detect both type IIA and IIB collagen with the use of the C-propeptide assay [24].

It is hoped that the availability of assays to measure degradative, synthetic, and turnover products of cartilage matrix metabolism in body fluids offers opportunities to try and monitor cartilage turnover *in vivo*. Type II collagen is degraded by proteolytic enzymes secreted by chondrocytes and synoviocytes. The cleavage of the type II collagen triple helix by collagenases results in the generation of neoepitopes at cleavage sites. Since the initial cleavage that generates the neoepitope is followed by subsequent cleavage of the alpha chain, there is release of the epitope from the tissue [26]. Thus, an increase in these cleavage products can be detected *in vivo *by immunoassays with antibodies that recognize cleavage epitopes called COL2-3/4C_longmono _(also known as C2C) and specific for type II collagen [14], and collagenase cleavage epitopes called COL2-3/4C_short _or C1,2C, which detect cleavages of both type II and I collagens [15], have been generated and are markedly elevated in experimental OA [27]. These epitopes are generated in OA articular cartilage as shown by Billinghurst and colleagues [15]. However, our investigation suggests that they are not detectably elevated in patients at risk of cartilage loss, and thus their role in predicting OA progression in human MRI studies is questionable based on our data. The inability to detect an increase may be related to the number of joints involved in OA (joint load) compared with RA [19,28]. Alternatively, it may be because, in serum, we are unable to distinguish increases in pathology from normal turnover.

Some recent work on type II collagen has suggested that assays for type II collagen degradation, when used alone or in combination or with markers of collagen synthesis, can distinguish populations with knee OA which exhibit progression of joint damage from non-progressors [2,29-31]. The ratio of the type II collagen crosslinking C-telopeptide (CTX-II) to the amino propeptide of type IIA collagen [2] or the ratio of two collagenase-generated cleavage epitopes in the helical region (C1,2C to C2C) [3] each can potentially make this distinction. In one of these clinical studies, progression was identified by the increase in type II collagen cleavage products compared to a decrease in the propeptide marker of synthesis [2]. That study had a number of major limitations, including the control subjects having no radiographic evaluation, incomplete evaluation of OA subjects, and only 12 months of follow-up. However, the greater the distinction between increased degradation and decreased synthesis, the more progression was observed. In another unpublished study of progression [3], the relative amount of primary and secondary cleavage correlated with progression. Both C2C and C1,2C epitopes contain the cleavage site generated by collagenase. The greater the amount of the shorter epitope (C1,2C) containing the cleavage site, relative to the longer epitope (C2C), the greater the progression. This suggests that there is a difference in proteolysis linked to progression that leads to increased generation of the shorter C1,2C epitope. Our investigation did not corroborate these findings, potentially due to differences in study design.

In addition to type II collagen, the second main component of the extracellular matrix of articular cartilage is aggrecan, which, like collagen type II, is almost specific to this tissue. Aggrecan is a proteoglycan composed of a core protein to which GAG chains are covalently attached. The compressive stiffness of articular cartilage is a product of the hydration and swelling of aggrecan, embedded as macromolecular aggregates within the collagen fibrillar network. The monoclonal antibody CS 846 prepared for aggrecan reveals the presence of the largest apparently intact molecules that predominate in fetal cartilages but that are almost absent from healthy adult cartilage [32]. In OA, these larger molecules reappear and increase in amount with increased synthesis of this molecule [17] in synovial fluid and serum [33,34]. The 846 monoclonal antibody usually recognizes the epitope on the largest molecules and likely signifies the presence of more recently synthesized molecules [17]. We are unaware of previous human clinical studies that have evaluated this promising biomarker and its relation to knee OA progression. Based upon our investigation, its role in predicting progression in knee OA may be limited, although its early increase has been observed in serum in experimental dog OA [35].

Cartilage oligomeric protein is a pentameric protein of the thrombospondin family which can bind type I, II, and IX collagens [36]. It is synthesized by chondrocytes, synovial cells, and other cells of the skeleton. Its synthesis is increased in chondrocytes and in synovial cells when activated by proinflammatory cytokines [37]. Preliminary plain radiographic studies suggested that COMP may be a useful prognostic marker of disease progression in knee [4-6] and hip [7] OA, and longitudinal analysis of COMP may predict episodic or phasic progression of OA [38]. We were able to corroborate these findings in a knee MRI study, suggesting that this marker may be a useful means of identifying progressors, albeit the estimate was modest.

Some further limitations of this work, some of which are generic to the application of biomarkers, warrant mentioning. Age-related increases are commonly seen in biochemical markers and these may produce variation in both biomarker levels and cartilage loss [39]. Efforts were made to adjust for age in analyses. The BOKS study assessed the local structural changes in the knees only and in only one knee (not both). It may be that other studies that investigate the total body burden of OA, including other joint areas, may be able to detect an association in patients with symptomatic OA. Another potential explanation for our null findings in patients with symptomatic OA is that we have insufficient power. It is further possible that other biomarkers that we did not measure could have a relation to MRI cartilage loss such as matrix metalloproteinase-3, high sensitive C-reactive protein, hyaluronan, CTX-I, keratan sulphate, matrillin-1, and cartilage intermediate layer protein. In addition, longitudinal analyses of biomarker change would be interesting to explore.

Another potential explanation for the lack of association found relates to limitations with the endpoint, namely cartilage loss on MRI. This was measured semiquantitatively with inherent potential observer bias and possible measurement error. Nonetheless, a number of studies have found plausible biologic associations with cartilage loss on MRI in this dataset, including relations to alignment [40], bone marrow lesions [41], and meniscal abnormalities [42]. Thus, any lack of ability to detect a strong association between biomarkers and MRI is unlikely to be a result of limitations in the MRI variable.

## Conclusion

With the exception of COMP, if changes in cartilage turnover in patients with symptomatic knee OA are associated with cartilage loss, they do not appear to affect systemic biomarker levels. Where there are other markers such as alignment and bone marrow lesions that are potent predictors of progression, we would not advocate one-time measurement of biochemical markers to predict MRI progression in patients with symptomatic knee OA, with the possible exception of COMP.

## Abbreviations

BMI = body mass index; BOKS = Boston Osteoarthritis of the Knee Study; C2C (also called Col2:3/4C_long_) = collagenase cleavage of triple-helical type II collagen; CI = confidence interval; Col2CTx = crosslinked peptides from the C-telopeptide domain of type II collagen; COMP = cartilage oligomeric matrix protein; CPII = C-propeptide of type II collagen; ELISA = enzyme-linked immunosorbent assay; FOV = field of view; K&L = Kellgren & Lawrence; MRI = magnetic resonance imaging; OA = osteoarthritis; TE = time to echo; TR = repetition time; WORMS = Whole Organ Magnetic Resonance Imaging Score.

## Competing interests

The authors declare that they have no competing interests.

## Authors' contributions

DH conceived of the study, participated in its design and coordination, and drafted the manuscript. JL and ML carried out the statistical analyses. JD carried out the assays. AG and DG read and interpreted the MRIs. DB, MN, RP, DE, and DF participated in the design of the study. All authors read and approved the final manuscript.
